# A Novel Bioswitchable miRNA Mimic Delivery System: Therapeutic Strategies Upgraded from Tetrahedral Framework Nucleic Acid System for Fibrotic Disease Treatment and Pyroptosis Pathway Inhibition

**DOI:** 10.1002/advs.202305622

**Published:** 2023-11-20

**Authors:** Yueying Jiang, Songhang Li, Ruijianghan Shi, Wumeng Yin, Weitong Lv, Taoran Tian, Yunfeng Lin

**Affiliations:** ^1^ State Key Laboratory of Oral Diseases National Center for Stomatology National Clinical Research Center for Oral Diseases West China Hospital of Stomatology Sichuan University Chengdu Sichuan 610041 China; ^2^ Sichuan Provincial Engineering Research Center of Oral Biomaterials Sichuan University Chengdu Sichuan 610041 China

**Keywords:** bioswitchable miR inhibitor delivery system, gene delivery, miRNAs, pyroptosis, skin fibrosis, tetrahedral framework nucleic acids

## Abstract

There has been considerable interest in gene vectors and their role in regulating cellular activities and treating diseases since the advent of nucleic acid drugs. MicroRNA (miR) therapeutic strategies are research hotspots as they regulate gene expression post‐transcriptionally and treat a range of diseases. An original tetrahedral framework nucleic acid (tFNA) analog, a bioswitchable miR inhibitor delivery system (BiRDS) carrying miR inhibitors, is previously established; however, it remains unknown whether BiRDS can be equipped with miR mimics. Taking advantage of the transport capacity of tetrahedral framework nucleic acid (tFNA) and upgrading it further, the treatment outcomes of a traditional tFNA and BiRDS at different concentrations on TGF‐β‐ and bleomycin‐induced fibrosis simultaneously in vitro and in vivo are compared. An upgraded traditional tFNA is designed by successfully synthesizing a novel BiRDS, carrying a miR mimic, miR‐27a, for treating skin fibrosis and inhibiting the pyroptosis pathway, which exhibits stability and biocompatibility. BiRDS has three times higher efficiency in delivering miRNAs than the conventional tFNA with sticky ends. Moreover, BiRDS is more potent against fibrosis and pyroptosis‐related diseases than tFNAs. These findings indicate that the BiRDS can be applied as a drug delivery system for disease treatment.

## Graphical abstract

A novel bioswitchable miR mimic delivery system (BiRDS) carrying miR‐27a is designed to treat skin fibrosis. This system is three times more efficient than the conventional tetrahedral framework nucleic acid transport (tfNA) system at delivering miRNAs, evidenced by BiRDS being more effective against fibrosis and pyroptosis‐related diseases compared to the tfNA system.

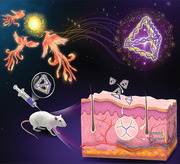

## 1 Introduction

The development of nanoscale nucleic acid drugs is rapidly advancing in the field of gene delivery. Gene vectors, vehicles that introduce genetic materials or gene‐editing devices into cells or tissues to modulate therapeutic proteins,^[^ ^1^ ^]^ have been extensively explored by scientists. The development of this field has raised the possibility of developing efficient nucleic acid drugs and treatments for previously incurable diseases.^[^ ^2^ ^]^ Compared with typically delivered linear or circularized nude single‐stranded (ss) DNA or RNA, tetrahedral framework nucleic acids (tFNAs) are innovative because of their accessibility, biocompatibility, tissue regeneration ability, and ability to deliver insoluble molecules and oligonucleotides.^[^ ^3^ ^]^ Lin et al.^[^ ^4^ ^]^ reported a self‐assembled tetrahedral framework nucleic acid with a wide range of functions in disease treatment as an anti‐inflammatory agent, immunomodulator, and a remarkable vector for enhancing the cellular delivery of small non‐water‐soluble molecules and oligonucleotides.

MicroRNAs (miRNAs, miRs), a large family of small, ≈21‐nucleotide‐long noncoding RNAs, can extensively interact with large subsets of target gene spectra.^[^ ^5^ ^]^ MiRNAs are predicted to regulate the activity of more than 60% of all protein‐coding genes and participate in regulating almost all complex genetic networks and cellular signaling cascades. Even a single miR can regulate a broad spectrum of targets and is consequently efficient and attractive.^[^ ^6^ ^]^ Hence, miRs may function as pivotal therapeutic tools for various disease phenotypes.^[^ ^7^ ^]^ Generally, the miRNA therapeutic strategy is based on suppressing pathogenic miRs and restoring palliative miRs, which includes miR sponges, miR masking, locked nucleic acids, and so on.^[^ ^8^ ^]^ However, the impossibility of the passive diffusion of miRs through the lipid membrane and the absence of a foolproof miR‐delivering system are the remaining hurdles in the preclinical stages of miR therapeutics, owing to the inherent biosafety issues and skeptical transfection efficiency from the present nonviral and viral vectors.^[^ ^9^ ^]^ Intensive studies are expected to elucidate an optimized, cost‐effective miRNA delivery system with a high efficacy and low toxicity to deliver the exogenous miRNAs into cells and integrate with the genome to execute their proper functions.^[^ ^10^ ^]^

The development of nanoscale nucleic acid drugs is rapidly advancing in the field of gene delivery. Gene vectors that introduce genetic materials or gene‐editing devices into cells or tissues to modulate therapeutic proteins^[^ ^1^ ^]^ have been extensively explored. The development of this field has raised the possibility of developing efficient nucleic acid drugs and treatments for previously incurable diseases.^[^ ^2a^ ^]^ TFNAs are innovative because of their accessibility, biocompatibility, tissue regeneration ability, and ability to deliver insoluble molecules and oligonucleotides.^[^ ^3^, ^4^, ^11^ ^]^ It can exhibit notable anti‐inflammatory and antifibrosis effects in the skin and lung fibrosis model,^[^ ^12^ ^]^ and inhibit the pyroptosis pathway in vitro and in vivo.^[^ ^13^ ^]^ This novel nanoscale nucleic acid material can smoothly permeate through the lipid bilayer via the caveolin mediated endocytic pathway; however, the traditional tFNA structures with sticky ends are still facing some challenges in the delivery process because of the instability in complex serum circumstances and poorer penetration ability.^[^ ^5^, ^14^ ^]^

The latest developments in upgraded tFNAs today have led to the third generation of dynamic drug delivery vectors with biological switches, before that tFNAs were used as powerful tissue regeneration agents and static drug delivery vectors in the two previous versions. Inspired by the sun and immortal bird gold ornaments, Li et al.^[^ ^15^ ^]^ first reported an original tetrahedral framework nucleic acid analog, a BiRDS carrying miR inhibitors, which exhibits remarkable delivery specificity without changing the original structure and has shorter synthesis period. However, whether miR mimics can be equipped still needs to be researched, so we have attempted to design a novel BiRDS system for delivering miR mimics to deepen our collective knowledge. To further confirm the successful fabrication and superior treatment ability of BiRDS, a miR‐27a mimic was applied to skin fibrosis and pyroptosis pathway‐related disease models.

Fibrosis is triggered by physiological repair mechanisms, including chronic inflammatory responses and connective tissue regeneration, including collagen I proteoglycan, fibronectin, and elastin.^[^ ^16^ ^]^ Connective tissues and organ structures are damaged with the continuous progression of organ fibrous, and their functions are disrupted, thus leading to disability and death from many diseases. The skin is the primary barrier between the human body and the external environment and is susceptible to various pathogenic molecules, including NOD‐, LRR‐, and pyrin domain‐containing protein 3 (NLRP3)‐mediated pyroptosis pathways.^[^ ^17^ ^]^ Pyroptosis is a novel pathway of programmed cell death different from apoptosis and participates in a wide range of cellular activities, such as cell swelling, osmotic lysis, and the release of proinflammatory cytokines.^[^ ^18^ ^]^ Pyroptosis substantially influences the process of fibrosis, especially in the liver, lungs, and heart; this facilitates its use as a potential target in treating inflammation‐related diseases. In the presence of chronic inflammatory stimuli, the skin sustains irreversible fibrosis, and severe contractures, which inevitably affect joint mobility and esthetic appearance. In our previous study, we revealed a relationship between pyroptosis and skin fibrosis and found that tFNAs negatively regulate the pyroptosis pathway in every link. However, considering the crucial crosstalk between a variety of chronic or acute diseases, pyroptosis is still worth studying to establish a solid foundation for a novel approach.^[^ ^19^ ^]^ To further improve the results, miR‐27a was adopted owing to its inhibitory effect on pyroptosis and skin fibrosis.^[^ ^20^ ^]^ MiR‐27a is a key influencing factor in NLRP3‐mediated pyroptosis and effectively treats systemic sclerosis, featuring diffuse skin and lung fibrosis.^[^ ^21^ ^]^ As mentioned previously, we inserted the miR‐27a sequence and designed four DNA/RNA mixed single strands for BiRDS instead of placing a double‐stranded miR sequence on the apex or side arms of tFNA. The conventional tetrahedral structure, with a smaller size than the linear cohesive end joint, and synthetic simplicity of BiRDS increase the efficiency of the transportation vehicle and stability for drug delivery compared to second‐generation tFNAs.

## 2 Results and Discussion

### 2.1 Design, Generation, and Characteristics of BiRDS‐miR27a and tFNA

**Figure 1:**
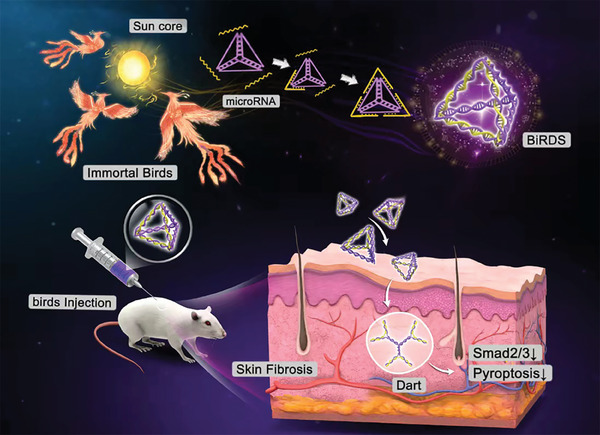
Successful synthesis of bioswitchable miRNA mimic delivery system (BiRDS) and inhibition of skin fibrosis. Three miRNA molecules formed a tetrahedral structure with nucleic acid core based on Watson‐Crick base pairing rules. Under the action of RNase H, BiRDS is converted to darts and released miRNA in cells and skin tissues. It protected miRNA mimics, reduced skin thickness, maintained epithelial polarity, and inhibited smad and pyroptosis pathway.

**Figure 2:**
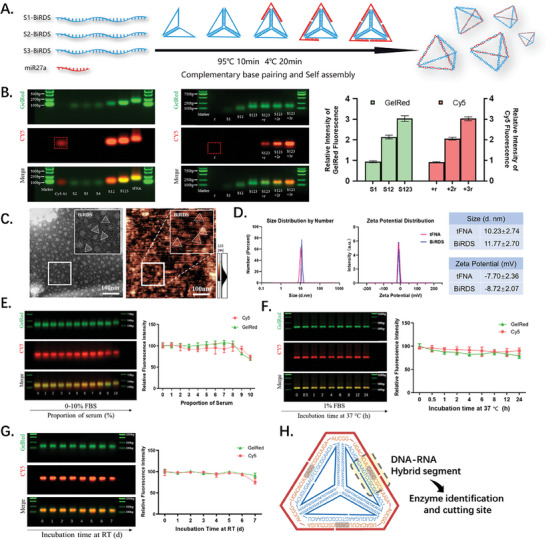
Synthesis, verification, characteristics, and stability of BiRDS. A) BiRDS were synthesized with one‐pot annealing, composing of “sun core” (inner blue kernel) and “immortal birds” (outer red wings). B) Agarose gel electrophoresis experiments illustrated the successful package of tFNA and BiRDS. The proportional growth of fluorescence intensity proved the stepwise polymer of the materials. The nude miR‐27a without the protection of sun core only exhibited weak fluorescence. C) The detection by transmission electron microscope and atomic force microscope showed the tetrahedral shape of tFNA and BiRDS, with the same diameter order of magnitude as data in Figure 2D. Scale bars are 100 nm. D) Size and Zeta potential distribution of BiRDS and tFNA by number, which demonstrated the nanoscale diameters and negative electric charge of materials. E,F) BiRDS exhibited excellent stability when reacting with 0%–10% FBS for 2 h under 37 °C. It also maintained prominent stability in 1% FBS for at least 24 h. G) BiRDS has reliable storage stability at room temperature (25 °C) and hardly breaks down in 7 days. H) Enzyme can specifically recognize DNA–RNA hybrid identification and cut side arms of BiRDS. Data are presented as mean ± standard deviation (SD) (*n* = 3).

In this study, we designed a bioswitchable vehicle that can enter cells and tissues while carrying miR mimics (**Figure** **1**). Three single‐stranded DNAs/RNAs (S1–3‐BiRDS) with specific nucleic acid sequences based on Watson–Crick base pairing comprise the “sun core,” and three miR‐27a mimics represent the “immortal birds” (**Figure** **2**A). Compared with our previously reported delivery system with sticky ends, which required over 50 min to synthesize,^[^ ^12^ ^]^ BiRDS can be fabricated in the same manner as tFNA through one‐pot annealing for 30 min. The successful synthesis of tFNA and BiRDS was confirmed by agarose gel electrophoresis, which revealed the molecular weights of the intermediate products, tFNA and BiRDS, from 30 to 180 bp (Figure 2B). Notably, after the same electrophoresis time, the Cy5 fluorescence intensity of the single strands was weaker than that loaded on the trestle, indicating that the overall structure would guarantee the stability of single strands more than the nude modalities (in the red dotted boxes). Single strands of miR27a proportionally adhered to the “Sun core” and showed the same fluorescence growth trend. Transmission Electron Microscope and atomic force microscopy (AFM) morphology analyses of tFNA and BiRDS revealed triangular nanoscale particles with a diameter of 10 nm (Figure 2C; Figure S1, Supporting Information), which was consistent with the size distribution determined by dynamic light scattering (Figure 2D). The tFNA and BiRDS were both negatively charged, with average zeta potentials of −7.70 and −8.72 mV, respectively. This feature facilitates the repulsion of ROS and the reaction with certain functional proteins, which is also the basic principle of electrophoretic separation.

### 2.2 Stability of tFNA and BiRDS

We incubated BiRDS with fetal bovine serum (FBS) (1–10%) for 2 h at 37 °C to simulate the in vivo environment and ideal growth conditions for cells. The fluorescence signal of Cy5 detected by AGE indicated that the BiRDS was minimally broken down in the presence of FBS (8%). The BiRDS retained 70% of the content of the nonserum group with a further increase in the FBS concentration to 10% (Figure 2E). In the in vitro model for skin fibrosis, we incubated HaCaT cells with BiRDS in FBS (1%) to simulate the cultivation environment and ensure that the BiRDS could efficiently enter the cells within a certain period. After 24 h, the fluorescence intensity of Cy5 remained at 85% of that at 0 h (Figure 2F). This guaranteed that the BiRDS retained its original form within 24 h, thus providing a reliable measurement of the number of particles entering the cells.

Storage conditions of biologics are extremely strict to preserve their efficacy; these conditions impose a high health cost on cryopreservation during cold chain logistics. BiRDS demonstrated extremely high stability at room temperature (25 °C) for at least 7 days and maintained at least 75% vitality of miR‐27a (Figure 2G), which is conducive to its application in complex environments and depressed areas. This is a reliable and convenient drug delivery system with substantial health benefits.

In the structure of BiRDS, there are three DNA‐RNA hybridized domains (containing hydrogen bonds) in the side arms, which enzyme can recognize and cut (Figure 2H). BiRDS exhibited excellent stability when incubated with RNase H from 12.5 to 200 U mL^−1^ (Figure S3, Supporting Information).

### 2.3 BiRDS Exhibited High Permeability and Promoted Cell Proliferation

**Figure 3:**
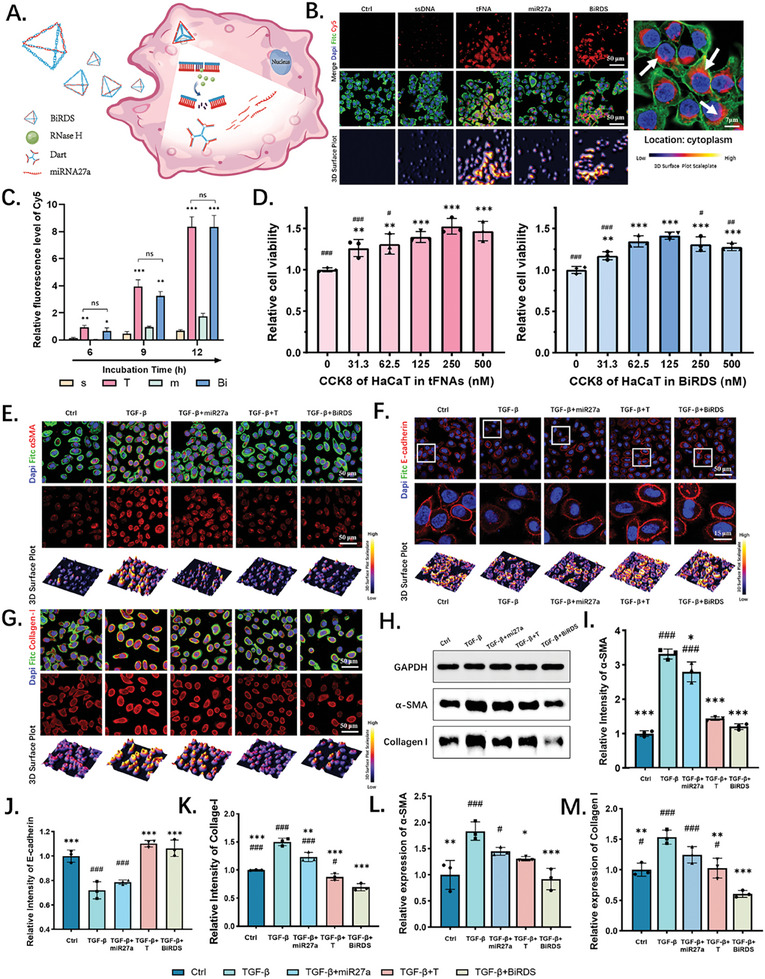
Cellular uptake of BiRDS and tFNA, cell proliferation, and effects on epithelial–mesenchymal transition (EMT) inhibition. A) The hybridization regions of BiRDS can hydrolyze in the presence of Rnase H. Each BiRDS molecule converted to a dart and released three miRs in cytosol. B) BiRDS and tFNA were largely uptaken into HaCaT cells after incubated for 12 h. Cy5 fluorescence signals were located at cytoplasm near the nucleus. C) Relative intensity of Cy5 labeling single strands, tFNA and BiRDS entering cells. D) Cell viability results via CCK8 experiments showed tFNA and BiRDS have positive effects on proliferation of HaCaT cells. E,I) Immunofluorescence (IF) results and 3D surface plots of αSMA expression after cells treated with TGF‐β, and its data analysis. F,J) IF staining and its statistical analysis of E‐cadherin. G,K) IF staining and its statistical analysis of Collagen‐I. H) Westernblot (WB) results were for testing protein expressions of αSMA and Collagen I, taking GAPDH as the internal standard. L,M) Data analysis of WB results in (H). Scale bars are 50 or 7 µm as labeled at the corner of images. Data are presented as mean ± standard deviation (SD) (*n* = 3). Statistical analysis: single tailed <0.05, double tailed <0.01, triplex tailed <0.001. * means “compared with the target in TGF‐β group”, and # means “compared with the target in BiRDS group”.

Interactions between BiRDS and HaCaT cells are essential for suppressing fibrosis. BiRDS enters the complex intracellular environment via a caveolin‐mediated endocytic pathway and is targeted by RNase H. The 3D structure of BiRDS was then converted into a 2D plane structure, and the “sun core” was stretched out to form a dart with three miR molecules. Furthermore, miR‐27a molecules were released into the cytoplasm to perform diverse functions (**Figure** **3**A). To compare the cellular uptake efficiency of BiRDS with that of tFNAs and the relevant single strands, we used immunofluorescence to detect the uptake of mi27a by inserting 250 nm fluorescent tFNA and BiRDS, as well as fluorescent single strands with identical Cy5 concentrations. As shown in Figure 3B, the BiRDS was located in the cytoplasm in the same manner and exhibited almost the same uptake efficiency as the tFNAs. After incubation with HaCaT cells for 12 h, the fluorescence intensity of Cy5 in the BiRDS and tFNA groups remained at the same level and showed no statistical differences, although it seemed that BiRDS entered the cells slightly later than tFNA (Figure 3C). Both showed high cell permeability.

As previously reported,^[^ ^22^ ^]^ after cells acquire tFNAs, this intuitive feature promotes cell proliferation. In this study, we utilized CCK8 experiments to detect cell proliferation and viability. After being incubated with different concentrations of tFNA and BiRDS ranging from 31.3 to 500 nm, the HaCaT cells showed an improved proliferation capacity, which suggested the nontoxicity of the two drugs (Figure 3D). Additionally, the tFNA and BiRDS showed the strongest ability to enhance cell viability at concentrations of 250 and 125 nm, respectively. Accordingly, we selected these as the optimal concentrations for subsequent experiments.

### 2.4 BiRDS Inhibits TGF‐β Induced Epithelial‐Mesenchymal Transition and Contributes to Maintaining Epithelial Stability In Vitro

Tissues contribute to resilience and strength by maintaining the extracellular matrix (ECM) secreted by mesenchymal cells and other matrix components.^[^ ^23^ ^]^ Overexpressed TGF‐β will promote epithelial cells to develop toward fibroblasts, known as an epithelial‐mesenchymal transition (EMT).^[^ ^24^ ^]^ Herein, we used TGF‐β as the actuating factor to establish an in vitro fibrosis model establishment.^[^ ^25^ ^]^ After adding 5 ng mL^−1^ TGF‐β to the culture medium for 24 h, α‐SMA, a mesenchymal marker, was upregulated by 3.2 times. However, it was reversed upon exposure to 250 nm tFNAs and 125 nm BiRDS. Compared with the tFNA group, the α‐SMA expression level was lower, and BiRDS restored the degree of EMT to almost the same level as in the control group (Figure 3E,H,I,L).

Epithelial (E)‐cadherin, known as a “cell adhesive” glycoprotein, is a single‐span transmembrane protein and is responsible for cell‐cell crosstalk and maintaining the polarity and integrity of epithelial cells.^[^ ^26^ ^]^ E‐cadherin silencing has long been considered a key actuating factor in influencing epithelial cells to detach from each other, causing a drastic loss in cell polarity.^[^ ^27^ ^]^ This factor can be regulated in terms of content or location by various fibrotic cytokines, including TGF‐β. In immunofluorescence (IF) tests, we observed that following the 5 ng mL^−1^ TGF‐β treatment, the fluorescence signal of E‐cadherin became weaker and blurred, suggesting its downregulation and mal‐positioning owing to EMT (Figure 3F,J). These changes were reversed following the treatment with tFNA and BiRDS. The protein returned to the membrane and showed sharper and slightly lighter fluorescence signals than the control group, contributing to the maintenance of HaCaT cell polarity and stability.

### 2.5 BiRDS Reduced Expressions of Matrix Proteins and Inhibited Smad 2/3 Pathway

**Figure 4:**
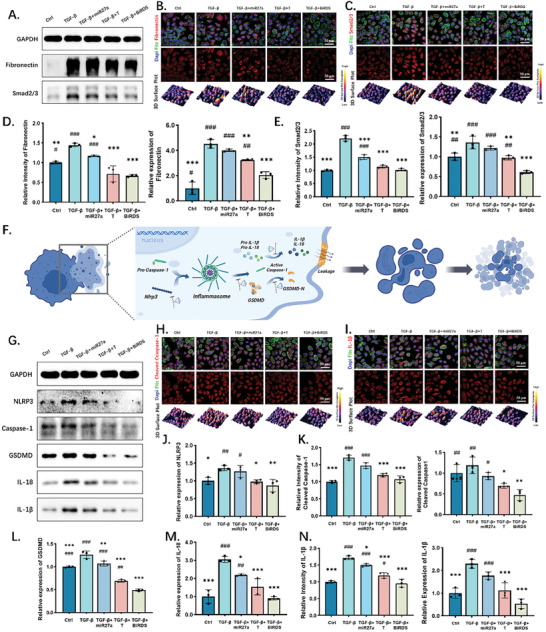
BiRDS inhibited Smad2/3 and pyroptosis pathways. A) WB results of fibronectin and Smad2/3 for analyzing protein expression levels. B,C) IF images and 3D surface plots of fibronectin and Smad2/3. D,E) Statistical analysis of WB and IF experiments in (A–C). F) Schematic illustration of pyroptosis pathway and BiRDS effects on different molecules and phases, from cell rupture to complete integration. G) BiRDS and tFNA inhibited pyroptosis pathway‐related proteins in WB results. H,I) IF images and 3D surface plots of Cleaved caspase‐1 and IL‐1β. J–N) Semi‐quantitative analysis of pyroptosis‐related protein expression in WB and IF experiments. All scale bars are 50 µm or 15 µm, as labeled at the corner of images. Statistical analysis: single‐tailed <0.05, double‐tailed <0.01, triple‐tailed <0.001. * represents the “compared with target in TGF‐β group,” and # represents the “compared with target in BiRDS group”.

In chronic fibrosis disorders, TGF‐β promotes the mesenchymal transformation of epithelial cells and their transition to myofibroblast‐like cells. In the advanced stages, the ECM, including collagen I, fibronectin, elastin, and proteoglycans, is increased because of increased fibroblast synthesis and decreased degradation.^[^ ^28^ ^]^ In our skin fibrosis model, 5 ng mL^−1^ of TGF‐β promoted the expressions of collagen I (Figure 3G,H,L,M) and fibronectin (**Figure** **4**A,B,D) in HaCaT cells by 1.6 and 4.3 times, respectively. Although the 250 nm tFNA primarily reverses the pathogenic synthesis of these two proteins as previously reported and miR‐27a molecules of equal concentrations exert their influence through accessing cells, BiRDS, as a more potent version of tFNA and a protectant for delivering miR‐27a, achieved a superior inhibitive effect at a low concentration of 125 nm.

TGF‐β is closely associated with fibrogenesis and is fundamental in regulating the ECM gene expression. Its downstream molecules, including Smad2/3, serve as basic and pivotal mediators in fibrogenesis and are an effective target in preventing tissue fibrosis.^[^ ^29^ ^]^ According to the western blotting (WB) and IF results (Figure 4A,C,E), Smad2/3 was downregulated when HaCaT cells were simultaneously treated with tFNAs and BiRDS. Although the tFNAs exhibited a potent treatment efficacy, at almost the same level as the control group, the BiRDS further strengthened the inhibitory reaction and optimized the results. These results demonstrated BiRDS is a more powerful nanoscale therapeutic agent for suppressing the TGF‐β/Smad pathway and treating skin fibrotic diseases.

During the three overlapping but distinct stages of wound repair (inflammation, new tissue formation, and remodeling),^[^ ^30^ ^]^ the tFNA clan members have shown superior therapeutic effects at every stage, and this newly upgraded dynamic biological switching material has further enhanced its efficacy.

### 2.6 BiRDS Carrying miR‐27a Suppressed NLRP3‐Mediated Pyroptosis in Skin Fibrosis Model

Briefly, the canonical pyroptosis pathway recruits NLRP3 and forms inflammasomes with pro‐caspase‐1. The active caspase‐1 promotes the cleavage of GSDMD and the cavity formation, which facilitates the leakage of mature IL‐18 and IL‐1β (Figure 4F). Following miR‐27a treatment, the NLRP3 expression was downregulated compared with the TGF‐β group (Figure 4G,J). Although tFNA showed a potent inhibitory effect on the pyroptosis pathway, BiRDS more strongly suppressed the downstream pathway proteins because of the interaction with miR‐27a (Figure 4H,I). The unique structure of BiRDS serves as a delivery vehicle for transiting miR‐27a into cells and preserves the stability of single strands, even if the same concentration of miRNA is added. In the BiRDS group, almost all the pyroptosis proteins were restrained to the level in the control group. By contrast, GSDMD, caspase‐1, and IL‐1β reached a lower line in the WB (Figure 4J–N), demonstrating that tFNA and BiRDS are antipyroptotic agents with a high application potential. As the simultaneous activation of smad and pyroptosis pathway was discovered in fibrotic diseases,^[^ ^31^ ^]^ the combined inhibition effect by BiRDS could be the next hotpots.

### 2.7 BiRDS Reduced Fibers in the Epithelium and Maintained Epithelial Structures

**Figure 5:**
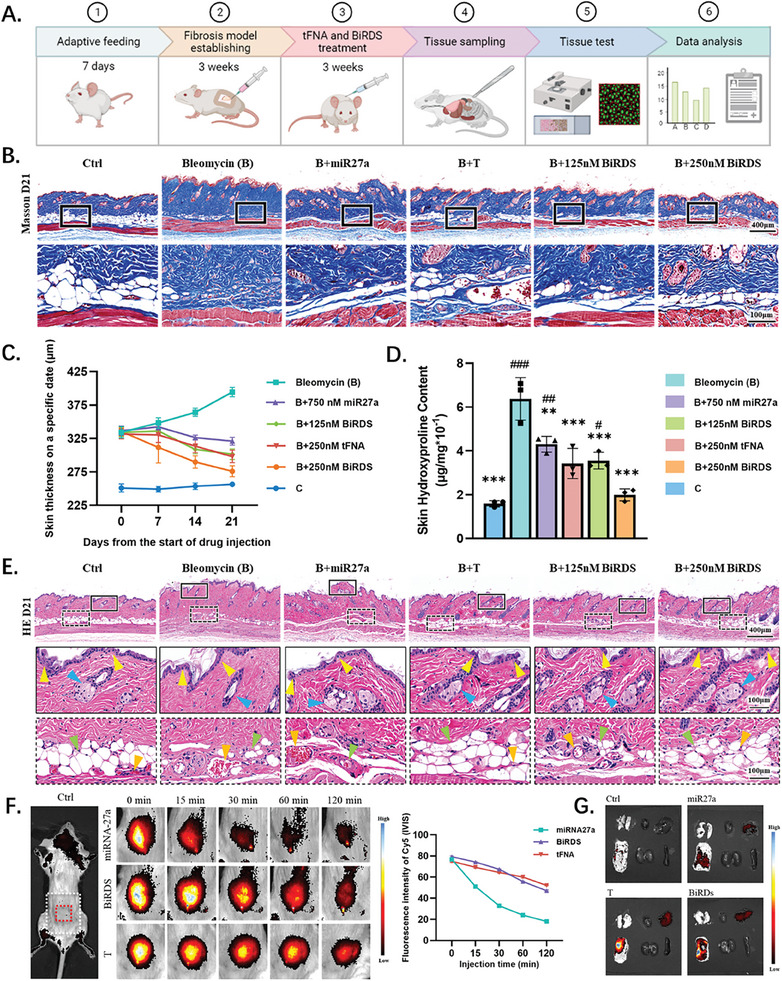
BiRDS inhibited skin fibrosis, maintained normal structures, and protected miR‐27a in vivo. A) Experimental scheme of in vivo experiments, including fibrosis model establishing, injection, molecule detection, and data analysis. B) Masson staining of skin tissues in each experiment group on day 21. C) Data analysis of skin thickness on days 0, 7, 14, and 21 showed 250 nm BiRDS had the most potent inhibition effect on skin fibrosis. D) Hydroxyproline content in each group was tested on day 21. E) HE‐staining images demonstrated change in skin tissues. Solid and dashed boxes magnified represented epithelial and subcutaneous tissue structures, respectively. (Yellow arrows: epithelial spikelike structure; blue arrows: glandular sebacea; green arrows: adipose tissue; yellow arrows: blood capillary.) F) Fluorescence signal intensity was detected within 120 min injection of the miR‐27a mimics, BiRDS and tFNA, and the semi‐quantitive analysis of fluorescence intensity was conducted. G) The tissues’ signals were detected at the end of 2 h. Scale bars are 400 or 100 µm, as labeled at the corner of images. Statistical analysis: single‐tailed <0.05, double‐tailed <0.01, triple‐tailed <0.001. *represents the “compared with target in bleomycin (B) group,” and # represents the “compared with target in 250 nm BiRDS group”.

During the final 3 weeks of treatment, we collected the middorsal skin tissues and viscera for testing every 7 days (**Figure** **5**A). Skin thickness often represents the primary endpoint of bleomycin‐induced fibrosis and can be specifically labeled with Masson's trichrome staining (Figure 5B). We measured the height from the epidermis to the subcutaneous tissue in three random non‐overlapping pictures for each group. The average measurements of dermal thickness in the bleomycin B) group increased from 334.2 to 394.4 µm as time went on (Figure 5C). The skin thickness decreased on day 21 in all treatment groups. BiRDS (125 nm) and tFNA (250 nm) exhibited the same effects as 750 nm miR‐27a. The thinnest skin tissue was observed in the 250 nm BiRDS group. The experimental results of the hydroxyproline assessment provided further verification of fibrosis content. Hydroxyproline is when the amino acid proline is hydroxylated during post‐translation and is largely restricted to collagen.^[^ ^32^ ^]^ Figure 5D illustrates the hydroxyproline content at the end of the experiment and shows a trend similar to that of skin thickness. The content from group B reached 0.64 µg mL^−1^, over three times higher than that of the control group, whereas 250 nm BiRDS reversed its effect to 0.20 µg mL^−1^ to the maximum.

During the injection period, the participants clearly felt that the skin became rougher and stiffer, and to observe the microstructure of the skin tissue, hematoxylin and eosin (H&E) staining was performed (Figure 5E). The solid and dashed boxes indicate the characteristic structures and changes in the epithelial and subcutaneous tissues, respectively. In the control group, we observed numerous dermal papillae, normal glandular structure, abundant subcutaneous adipose tissue, and capillary vessels.^[^ ^33^ ^]^ However, the drastic fibrotic process prompted the disappearance of glandular, adipose, and dermal papillae, and dilated blood vessels increasingly occupied additional positions in the subcutaneous tissue. The injection of 750 nm miR‐27a and 125 nm BiRDS restored only parts of the structure, whereas 250 nm tFNA and BiRDS exhibited prominent treatment efficacies.

### 2.8 BiRDS Maintained Stability of miR‐27a and Demonstrated Skin Targeting

To examine the metabolism in various organs after the subcutaneous injection of drugs, we tested the distribution with an IVIS Spectrum at certain points in 120 min (Figure 5F). Drugs of equal fluorescence intensity were injected into the mice with bleomycin‐induced fibrosis. A similar initial intensity was guaranteed immediately after the injection. After 15 min, the fluorescence intensity of miR‐27a decreased sharply and remained at a low level for the last 90 min. Conversely, a higher concentration of BiRDS was preserved in the skin tissue, and it slowly decreased to a lower level at 120 min but was still higher than that of nude miR‐27a (Figure 5G). The tFNAs degraded relatively uniformly compared to the other two drugs, probably because of their lower absorption rate. The BiRDS protects the stability of the DNA/RNA single strands and can target organs. We observed the lowest fluorescence in the miR‐27a group and the most intense fluorescence in the skin injected with BiRDS, indicating a strong stability and high skin‐targeting capacity. Moreover, a weak Cy5 fluorescence was observed in the liver after 120 min, indicating that the drugs were metabolized in the liver, as previously reported by our group. BiRDS showed no notable toxicity in the organs during our experiments and exhibited strong biocompatibility (Figure S2, Supporting Information).

### 2.9 BiRDS Inhibited TGF‐β/Smad Pathway in vivo

**Figure 6:**
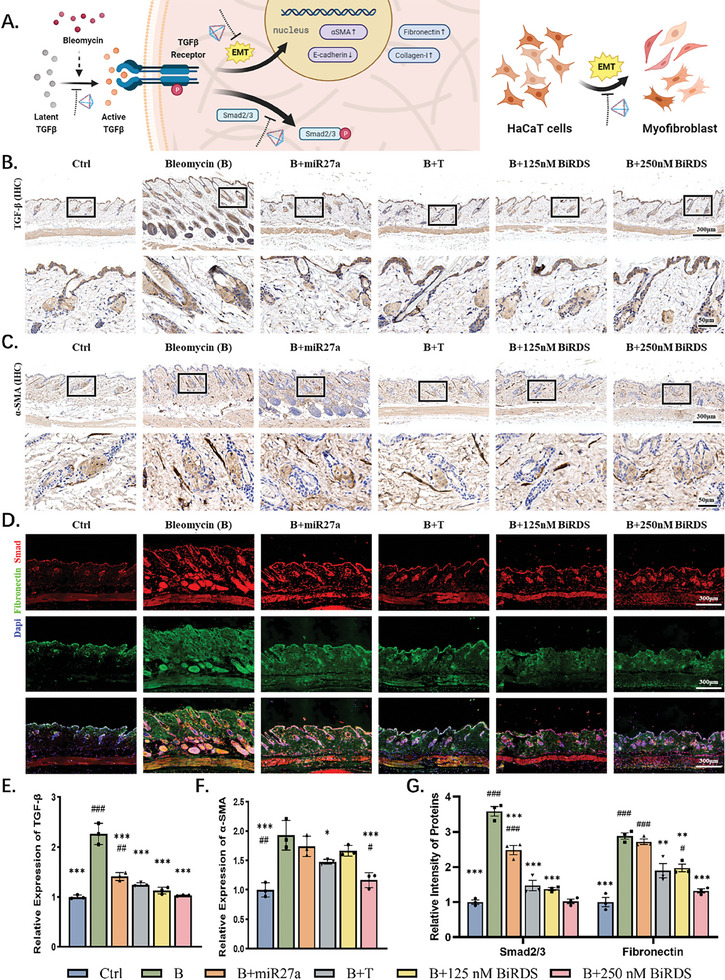
BiRDS inhibited skin fibrosis, maintained normal structures, and protected miR‐27a in vivo. A) Experimental scheme of in vivo experiments, including fibrosis model establishment, injection, molecule detection, and data analysis. B) Masson staining of skin tissues in each experiment group on day 21. C) Data analysis of skin thickness on days 0, 7, 14, and 21 showed 250 nm BiRDS had the most potent inhibition effect on skin fibrosis. D) Hydroxyproline content in each group was tested on day 21. E) Hematoxylin and eosin H,E) staining images demonstrated change in skin tissues. The solid and dashed boxes magnified represented epithelial structures and subcutaneous tissue structures, respectively. (Yellow arrows: epithelial spikelike structure; blue arrows: glandular sebacea; green arrows: adipose tissue; yellow arrows: blood capillary), F,G) Fluorescence signal intensity was detected within 120 min of injection of miR‐27a mimics, BiRDS, and tFNA, and tissue signals were detected after 2 h. Scale bars are 400 or 100 µm, as labeled at the corner of images. Statistical analysis: single‐tailed <0.05, double‐tailed <0.01, triple‐tailed <0.001. * represent the “compared with target in bleomycin (B) group,” and # represents the “compared with target in 250 nm BiRDS group”.

During the pathological process stimulated by bleomycin, latent TGF‐β molecules are initiated by a wide range of proteases, such as plasmin and metalloproteinase 9.^[^ ^34^ ^]^ TGF‐β is also a potent inducer of converting epithelial cells into myofibroblasts characterized by expressing α‐SMA (**Figure** **6**A). An increasing number of myofibroblast cells express excess ECM and redundant proteins are deficiently decomposed. The immunohistochemistry (IHC) results demonstrated that the TGF‐β content increased by ≈2.3 times in the bleomycin group (Figure 6B,E). Surprisingly, all the treatment groups demonstrated clear therapeutic effects. Nude miR‐27a reduced the TGF‐β content to 1.4 times that of the control group, whereas 250 nm BiRDS maintained it at a minimum, almost to the same degree as the control group. TFNA and 250 nm BiRDS had superior efficacies regarding α‐SMA inhibition; the latter group had the strongest effect, reaching a low level of 1.2 times that of the nonfibrosis group (Figure 6C,F).

The activation and release of TGF‐β, as well as its transgenic overexpression, promote fibrosis in various organs via a canonical signaling pathway that facilitates the transcription factor Smad2/3.^[^ ^34^ ^]^ As previously reported, increased levels of Smad2/3 enhance fibrosis in mice and patient samples, whereas the deletion of Smad genes has a protective and antifibrotic effect.^[^ ^35^ ^]^ Therefore, they are often therapeutic targets for fibrosis treatment. We used immunofluorescence to stain fibronectin and Smad2/3 simultaneously and photographed them under equal conditions. The inhibitory effects of 250 nm tFNA and 125 nm BiRDS on both proteins were almost identical, with 250 nm being the most powerful therapeutic agent (Figure 6D,G).

### 2.10 BiRDS Suppressed Pyroptosis‐Related Proteins and TNF‐α

**Figure 7:**
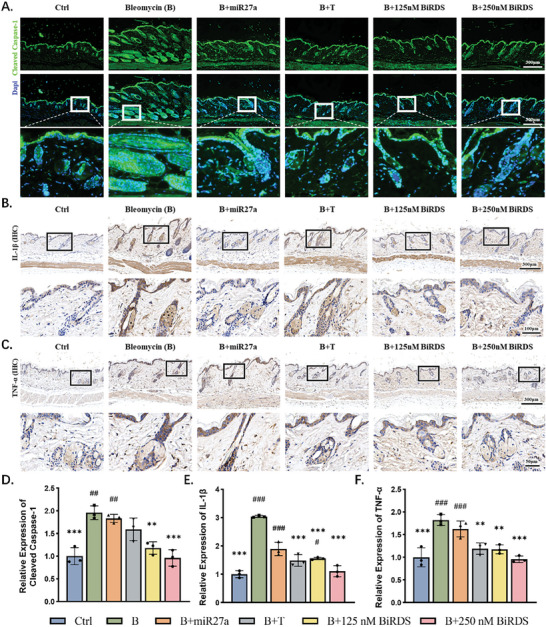
BiRDS inhibited bleomycin‐induced EMT and Smad pathway. A) Schematics illustrating TGF‐β activation, EMT, and Smad2/3 pathway. HaCaT cells converted to myofibroblasts under propulsion of EMT, which could be reversed by BiRDS. B,C) Immunohistochemical staining experiments of skin tissues were conducted to analyze protein levels of TGF‐β and αSMA, which were highly expressed in bleomycin groups and inhibited in group receiving 250 nm BiRDS. D) Double fluorescence staining images for evaluating expression levels of Smad and fibronectin (red: Smad; green: fibronectin). E,F) Semi‐quantitative analysis and statistical tests of proteins demonstrated in Figure 6B–D. Scale bars are 300 or 50 µm as labeled at the corner of images. Statistical analysis: single‐tailed <0.05, double‐tailed <0.01, triple‐tailed <0.001. * represents the “compared with target in bleomycin (B) group,” and # represents the “compared with target in 250 nm BiRDS group”.

Our in vitro experiments demonstrated that BiRDS carrying miR‐27a inhibited the pyroptosis pathway. To evaluate the role of BiRDS in regulating in vivo pathological processes, we conducted immunofluorescence and IHC experiments for cleaved caspase‐1 and IL‐1β and performed semi‐quantitative analyses of target proteins. The inflammation and fibrosis caused by bleomycin facilitated the strong expression of cleaved caspase‐1 in the hair follicles, sebaceous glands, and subcutaneous tissues (**Figure** **7**A). The hair follicles are a kind of complex mini‐organ constantly undergoing dynamic cycles of growth and regression throughout life and were proved to act as the “pathway” for small molecules penetrating tissues.^[^ ^13^ ^]^ We observed high fluorescence intensity changes of cleaved caspase‐1 in hair follicle tissue, demonstrating they are the key sites for the development of fibrosis and indirectly verified BiRDS carrying miRNA27a exhibited excellent skin‐targeting capacity.

The expression of the downstream inflammatory cytokine IL‐1β varied in the same manner (Figure 7B). Compared to tFNA, each BiRDS particle contained three miR‐27a molecules targeting NLRP3, which mediates the canonical pyroptosis pathway. As a result, BiRDS likely inhibited every step of the pyroptosis cascade pathway with the maximum efficiency among the four groups (Figure 7D,E). As the final link in the pyroptosis pathway, IL‐1β is the cytokine secreted from GSDMD holes and promotes the inflammatory response. In the bleomycin group, the expression level of IL‐1β rose three‐fold, and the BiRDS brought the level down to slightly higher than in the first group. Furthermore, we tested the expression of tumor necrosis factor‐alpha (TNF‐α), a key driver of fibrosis. This factor strongly influences the amplification and perpetuation of tissue damage and fibrosis. TNF‐α expression was also inhibited by 250 nm BiRDS most efficiently, which suggested BiRDS has a stronger efficacy than tFNA in inhibiting pyroptosis and other inflammatory factors, which ameliorated antipyroptotic and anti‐inflammatory activities and reduced fibrosis (Figure 7C,F).

## 3 Conclusion

In summary, we have designed and revealed a novel bioswitchable miR mimic delivery system (BiRDS) carrying miRNA‐27a mimics. This system offers several advantages, such as easy and time‐saving synthesis, storage stability, reliable biocompatibility, and biosafety. Cell permeability through endocytosis enables the particles to enter without the need for carriers and efficiently exert biological effects. Specifically, in this study, we observed that it effectively promoted cell proliferation, antifibrosis, antipyroptosis, anti‐inflammatory, nidus targeting, and protecting epithelial structures.

Regarding the structural design of the “sun core” and “immortal birds,” the overall tetrahedral structure protects the exposed miR molecules, increasing their stability and avoiding decomposition by enzymes in complex internal environments before they bind to target genes. Compared with the second‐generation tFNA carrying nucleic acid drugs through sticky ends, BiRDS can be synthesized in one pot within 30 min without an additional 1 h incubation at room temperature (25 °C). The retention of the original tetrahedral structure guaranteed the nonpolar structure of BiRDS and facilitated superior stability and permeability with a smaller diameter through multiple vertex attacks. Instead of the limitations of tFNA with sticky ends carrying only one miR,^[^ ^35^ ^]^ each BiRDS can carry three miRs and efficiently deliver them into cells and tissues, thereby reducing the drug concentration required for treatment.

In this study, we compared the biological effects of traditional tFNA in first‐ and third‐generation dynamic responsive delivery systems of BiRDS. The miRNAs were clearly delivered more efficiently with the assistance of BiRDS, and we selected miR‐27a to target the NLRP3‐mediated pyroptosis pathway. The therapeutic effects of nude miRs were weaker than those of the other two drugs because the exposed single RNA strands lost protection and quickly decomposed; the same was true in the cellular uptake experiment, with very few miR‐27a molecules entering the HaCaT cells and exerting effects. Nude miRNAs, tFNAs, and BiRDS have anti‐inflammatory and antioxidant effects and inhibit the Smad and pyroptosis pathways. However, during the cell experiments, the BiRDS at 125 nm had the strongest influence owing to the specific therapeutic effects of miRNAs, with half the concentration of tFNA. Furthermore, the in vitro and in vivo experiments conducted in this study indicate that BiRDS exhibits non‐toxicity, reliable biocompatibility, and biosafety. As a result, it holds promise for widespread utilization in the treatment of pyroptosis and fibrosis‐related diseases. Besides, smad‐mediated TGF‐β signaling can activate the NLRP3 inflammasome, while proteins of smad signaling pathways can directly interact with inflammasome components, further influencing pyroptotic outcomes.^[^ ^36^ ^]^ In our experiments, we verified the inhibition effects of BiRDS on the two pathways separately, but the interconnections between them may become the focus of our future research.

This study synthesized, applied, and compared first‐generation tFNA nanomaterials and an upgraded generation of dynamic bioswitchable delivery systems simultaneously. Although both drugs showed significant therapeutic effects, overall, BiRDS showed a higher efficacy versus tFNA, especially in the cell experiments, where the former yielded superior results at only half the concentration. Therefore, applying tFNA alone or combined with BiRDS for skin wound repair has extremely bright prospects and a high potential for clinical research and commercial cosmetology development.

Our experimental protocols could be optimized after confirming the successful synthesis of BiRDS and its application in pyroptosis‐related skin fibrosis. By redesigning the nucleic acid sequences of the “sun core” and “immortal birds,” this novel drug delivery system can simultaneously deliver up to three miRs of different clans; this function is practically impossible when three kinds of oligonucleotides are loaded on the side arm, because excessive types of single strands will greatly increase the probability of unexpected pairings, leading to a sharp rise in the design and experimental costs. This study not only focused on chronic diseases but also provided a research basis for acute disease treatment.^[^ ^37^ ^]^

## 4 Experimental Section

### Design and Generation of BiRDS and tFNA

The sequences of nucleotides in single DNA/RNA strands (ssDNA/ssRNA) followed the design based on the complementary pairing principle shown in Table S1 (Supporting Information). All ssDNA/ssRNAs were synthesized by Sangon Co., Ltd. (Shanghai, China). As in our previous research, to generate tFNAs, equimolar concentrations of four ssDNA (S1–4‐T, 1 µm) were added to TM buffer (10 mm Tris‐HCl and 50 mm MgCl_2_ dissolved in water, and hydrochloric acid was utilized to adjust the pH value to 8.0), after that the entire solution was heated to 95 °C for 10 min and immediately cooled at 4 °C for 20 min in a thermal cycler to complete the denaturation and assembling process. Equimolar concentrations of S1–3‐BiRDS were utilized to synthesize the BiRDS particles. Three times the concentration of miR‐27a strands was added to the tube to form the integrated BiRDS tetrahedral structure.

### Authentication and Characteristics of BiRDS and tFNA

To determine the successful synthesis of BiRDS and tFNA, agarose gel (2%) electrophoresis (AGE) dyed by GelRed was utilized with an electrophoresis apparatus (Bio‐Rad, USA) operating at 120 V for 30 min. To verify the tetrahedral nanostructures of the materials, their morphologies were observed using AFM (Shimadzu, Kyoto, Japan) and TEM (Hitachi Ltd., Tokyo, Japan). Sizes and zeta potentials were measured using a Zetasizer Nano ZS90 system (Malvern, United Kingdom).

### Serum Stability and RNase H Reaction of BiRDS and tFNA

To verify the serum stability and storage capacity at room temperature, the serum stability was tested under three reaction conditions, including FBS (Corning Inc., NY, USA) (0–10%) and different durations from 0.5 h to 7 days. Different concentrations of RNase I (Sangon Biotechnology, China) from 0 to 200 U mL^−1^ were added to the reaction system for 12 h at 25 °C. The final products were detected using AGE experiments conducted in 0.5× TAE buffer containing Mg^2+^ (5 mm). To ensure the stability of the final product during detection, we cooled the liquid medium in advance and placed it in ice baths. Gels were visualized using a gel‐and‐blot imaging system (Syngene, Bangalore, India).

### Cellular Uptake and Cell Treatment

HaCaT cells (BNCC, Henan, China) were cultured in RPMI‐1640 medium containing FBS (10%) and penicillin/streptomycin (1% (v/w) Hyclone, USA) in a humid incubation environment at 37 °C and CO_2_ (5%). Before the cells were incubated with the nucleic acid drugs, the FBS in the culture flasks was gradually decreased to 1% for 3 h to reduce the formation of the protein corona and prepare for the next operation. For the cellular uptake experiments, HaCaT cells seeded in confocal dishes were cultured in a medium containing nucleic acid drugs at different concentrations (Cy5‐tFNA 250 nm, Cy5‐BiRDS 250 nm, Cy5‐S1‐T 750 nm, Cy5‐miRNA‐27a 750 nm). After incubation for 4 h, the samples were fixed with 4% paraformaldehyde for 30 min, and DAPI and fluorescein isothiocyanate (FITC) were used to label the nucleus and cytoplasm, respectively. The sections were rinsed thrice with PBS for 5 min between adjacent steps. Finally, images were captured using a confocal laser microscope (FV3000, Olympus, Japan).

Except for the control group, the other four groups were treated with 5 ng mL^−1^ TGF‐β (transforming growth factor β) (MedChemExpress, Monmouth Junction, NJ, USA) for 24 h to build a skin fibrosis model. Simultaneously, the miR‐27a mimics, tFNAs, and BiRDS were added at concentrations of 375, 250, and 125 nm, respectively.

### Cell Viability Measurement

To examine the effect of nucleic acids on HaCaT cells, a cell counting kit‐8 (CCK8) assay was performed. Various concentrations (31.3, 62.5, 125, 250, and 500 nm) of tFNAs and BiRDS were incubated with cells in 96‐well plates for 24 h, after that CCK‐8 (10 µL) solution was added into the wells, and the absorbance was measured at 450 nm.

### Immunofluorescence (IF) Assessment

After the HaCaT cells were seeded on confocal dishes and the density approached 60%, the related modeling and treatments were conducted as described previously. The samples harvested were fixed by 4% paraformaldehyde for 30 min, 0.5% Triton X‐100 for 10 min, and 5% sheep serum for 1 h. Diluted primary antibodies of different concentrations were incubated at 4 °C overnight (α‐smooth muscle actin (α‐SMA), 1:500; fibronectin, 1:300; collagen I, 1:300; E‐cadherin, 1:500; Smad2/3, 1:300; Cleaved caspase‐1, 1:250; IL‐1β, 1:150), after that the corresponding fluorescent secondary antibodies, FITC and DAPI (Servicebio, Wuhan, China) were incubated with the samples for 1 h, 30 min, and 10 min, respectively. Similarly, the fluorescence signals were captured using a confocal laser microscope, whereas the fluorescence intensity determination of one protein selected an identical parameter.

### Establishment of Animal Model and Grouping

All animal experiments were performed in accordance with the Animal Ethics Committee of West China College of Stomatology, Sichuan University. Sixty BALB/c mice (6–8‐weeks old, 18–22 g) were purchased from Gempharmatech Co., Ltd. (Nanjing, China) and maintained in a 12/12 h light/dark cycle. They served as the fibrosis animal model and the specific model‐building method was in accordance with our previous study.^[^ ^15^, ^38^ ^]^ Briefly, after 7‐day adaptive feeding, mice were injected with bleomycin hydrochloride (B) (0.1 mL 0.5 mg mL^−1^) on a specific area of the dorsal skin every other day for 3 weeks. During the next three weeks, the mice were divided into six groups: control, B, B+miR27a, B+T, B+125 nm BiRDS, and B+250 nm BiRDS, and injected with nucleic acid drugs. The harvested viscera were used for tissue analyses.

### Skin Thickness and Tissue Pathology Analysis

The dehydrated and paraffin‐embedded tissues were sliced into 4‐µm sections. The skin was flattened and sliced longitudinally to observe the lesion site in the center of the tissue using an FSX100 microscope (Olympus, Tokyo, Japan). After Masson staining, the skin fibrosis was evaluated by measuring the vertical epithelial thickness of the blue fibrotic part. H&E staining was used to observe histological changes in the skin and viscera. The dried slices were immediately placed in xylene, dewaxed for 5–10 min, and placed in alcohol (100%–70%) until the alcohol was washed off with water. After staining with hematoxylin and eosin, the tissues were dehydrated. To facilitate the observation and preservation, neutral balsam was dripped onto the tissue and baked.

### IHC Analysis

To perform IHC, the remaining sample slices were cultured in diluted primary antibodies at 4 °C overnight, including mouse anti‐TGF‐β. A specific diluted anti‐rabbit secondary antibody (1:600, USA) was used at 37 °C for 20 min. After development, washing, dehydration, and mounting, the slices were imaged using an optical microscope.

### BiRDS and tFNA Distributions in vivo

To investigate the distribution, stability, and absorption rate of the injected drugs, we penetrated Cy5‐BiRDS (250 nM), Cy5‐tFNAs (250 nm, with Cy5 in S1–3) and Cy5‐mi27a (750 nm) and performed in vivo imaging experiments. After different time periods (0, 15, 30, 60, and 120 min), the mice were anesthetized with isoflurane and imaged using an in vivo imaging system (IVIS Spectrum, Shanghai, China). After 120 min, the mice were euthanized to isolate the organs, including the skin, heart, liver, spleen, lungs, and kidneys, which were simultaneously exposed.

### Ethical Statement

This study was approved by the Medical Ethics Committee of Sichuan University. The approval number is GB14925‐2010.

## Conflict of Interest

The authors declare that there is no conflict of interest.

## Supporting information

Supporting Information

## Data Availability

The data that support the findings of this study are available from the corresponding author upon reasonable request.
