# Supplementary material for: A Novel Bioswitchable miRNA Mimic Delivery System: Therapeutic Strategies Upgraded from Tetrahedral Framework Nucleic Acid System for Fibrotic Disease Treatment and Pyroptosis Pathway Inhibition
Source: Adv Sci (Weinh). 2023 Nov 20;11(1):2305622. doi: 10.1002/advs.202305622 (PMC10767442; doi:10.1002/advs.202305622)
Supplement: Supplementary file 1 — Supporting Information [file ADVS-11-2305622-s001.pdf]

ADVANCED SCIENCE
Open Access

# Supporting Information

for *Adv. Sci.*, DOI 10.1002/advs.202305622

A Novel Bioswitchable miRNA Mimic Delivery System: Therapeutic Strategies Upgraded from Tetrahedral Framework Nucleic Acid System for Fibrotic Disease Treatment and Pyroptosis Pathway Inhibition

*Yueying Jiang, Songhang Li, Ruijianghan Shi, Wumeng Yin, Weitong Lv, Taoran Tian* and Yunfeng Lin**

**Supporting Information**

**A Novel Bioswitchable MiRNA Mimic Delivery System: Therapeutic Strategies Upgraded from Tetrahedral Framework Nucleic Acid System for Fibrotic Disease Treatment and Pyroptosis Pathway Inhibition**

*Yueying Jiang[1], Songhang Li[1], Ruijianghan Shi[1], Wumeng Yin[1], Weitong Lv[1], Taoran Tian[1,*], Yunfeng Lin[1,2*]*

1. State Key Laboratory of Oral Diseases, National Center for Stomatology, National Clinical Research Center for Oral Diseases, West China Hospital of Stomatology, Sichuan University, Chengdu, Sichuan 610041, China

2. Sichuan Provincial Engineering Research Center of Oral Biomaterials, Chengdu, Sichuan 610041, China

E-mail: yunfenglin@scu.edu.cn; taoran.tian@scu.edu.cn

Supporting Information:

Table S1

Figure S1-S4

Method S1-S2

**Table S1** Base sequences of the single strands used to construct the BiRDS, tFNA and Cy5 labeled molecules.

**Table S1. Base sequences of the single strands used in the experiments**

| Strand | Direction | Base sequence (Uppercase letters: DNA, lowercase letters: RNA) |
|---|---|---|
| S1-T | 5′→3′ | ATTTATCACCCGCCATAGTAGACGTATCACCAGGCAGTTGAGACGAACATTCCTAAGTCTGAA |
| S2-T | 5′→3′ | ACATGCGAGGGTCCAATACCGACGATTACAGCTTGCTACACGATTCAGACTTAGGAATGTTCG |
| S3-T | 5′→3′ | ACTACTATGGCGGGTGATAAAACGTGTAGCAAGCTGTAATCGACGGGAAGAGCATGCCCATCC |
| S4-T | 5′→3′ | ACGGTATTGGACCCTCGCATGACTCAACTGCCTGGTGATACGAGGATGGGCATGCTCTTCCCG |
| Cy5-S1-T | 5′→3′ | Cy5-ATTTATCACCCGCCATAGTAGACGTATCACCAGGCAGTTGAGACGAACATTCCTAAGTCTGAA |
| Cy5-S2-T | 5′→3′ | Cy5-ACATGCGAGGGTCCAATACCGACGATTACAGCTTGCTACACGATTCAGACTTAGGAATGTTCG |
| Cy5-S3-T | 5′→3′ | Cy5-ACTACTATGGCGGGTGATAAAACGTGTAGCAAGCTGTAATCGACGGGAAGAGCATGCCCATCC |
| S1-BiRDS | 5′→3′ | aagugucaAGGATGCGCATGCTCTTGGCGACGGTATTGGACGGTCGCATGAucaaggcgCCTG |
| S2-BiRDS | 5′→3′ | aagugucaACATGCGACCGTCCAATACCGACGATTACAGCTTGCTACACGAucaaggcgCCTG |
| S3-BiRDS | 5′→3′ | aagugucaACGTGTAGCAAGCTGTAATCGACGCCAAGAGCATGCGCATCCAucaaggcgCCTG |
| miRNA-27a | 5′→3′ | cgccuugaaucggugacacuu |
| Cy5-miRNA-27a | 5′→3′ | Cy5-cgccuugaaucggugacacuu |

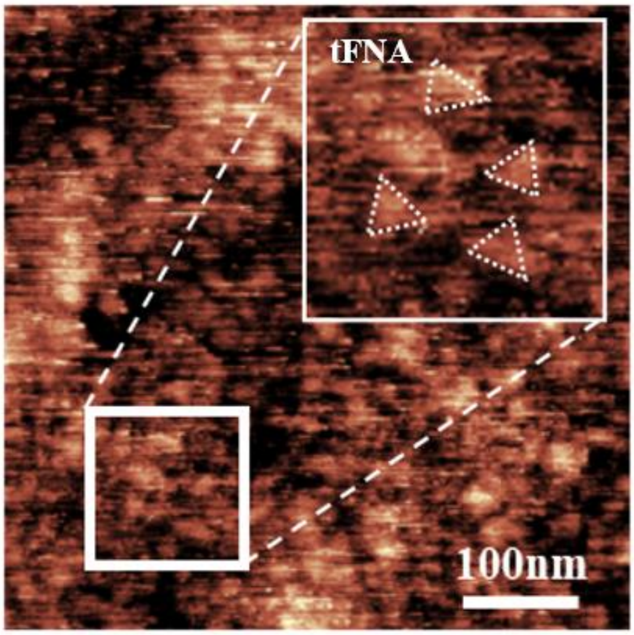


**Figure S1 Images of tFNA captured by Atom force microscope showed the shape of tFNAs and the diameter.**

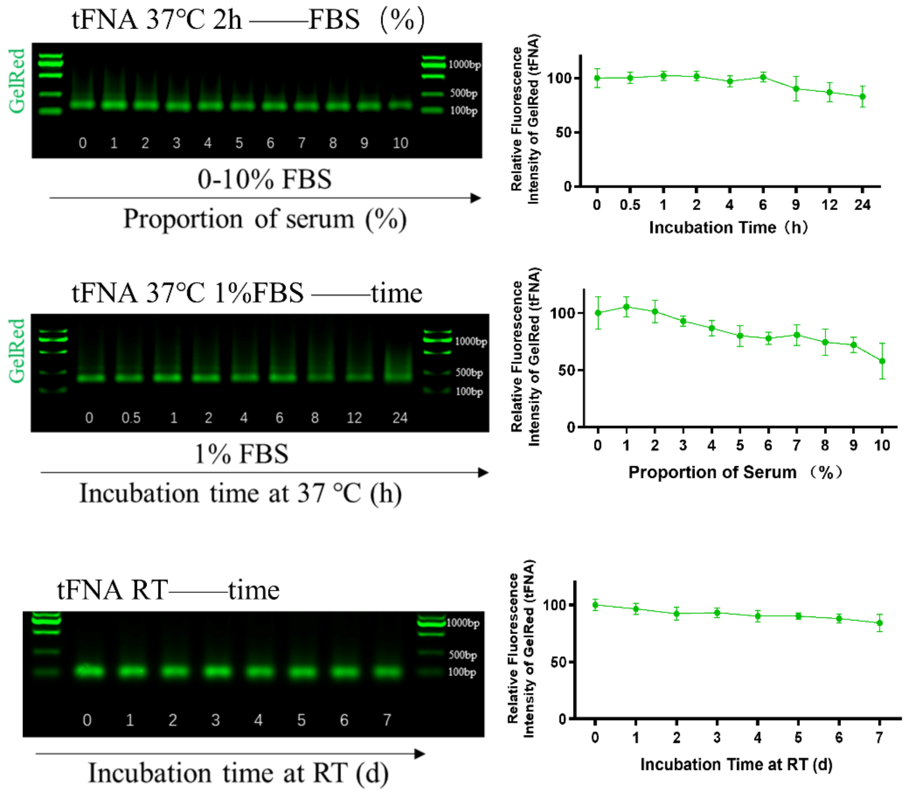


**Figure S2** TFNA showed excellent stability when incubated in 0–10% FBS conditions for 2h, as well as in 1% FBS within 24 h. Storage stability was also verified when tFNA was incubated in 7 days at room temperature (25 °C).

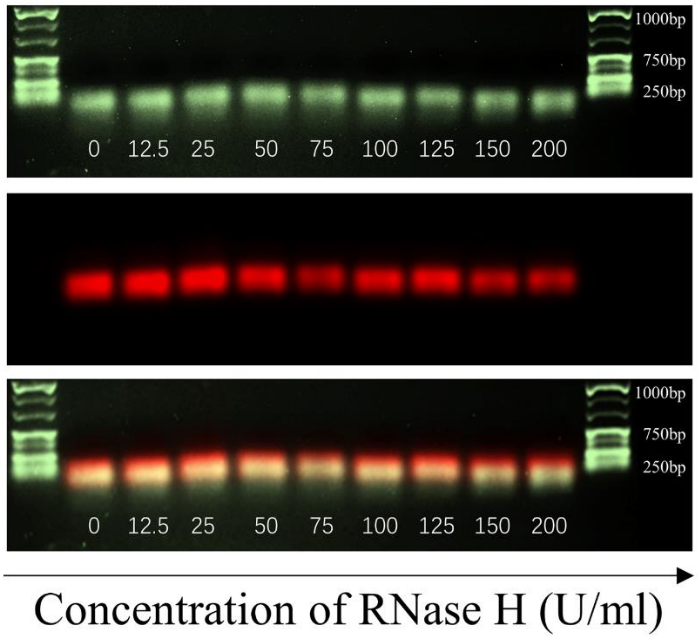


**Figure S3** BiRDS showed excellent stability when incubated in 0-200 U/ml RNase H, as well as in 1% FBS within 24 h.

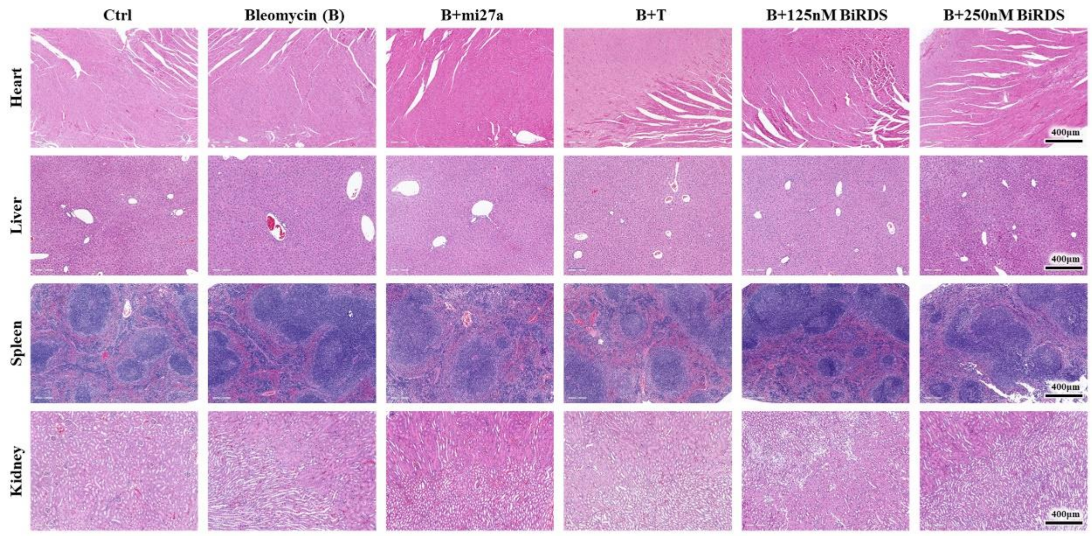


**Figure S4** The hematoxylin and eosin (H&E) staining of hearts, livers, spleens and kidneys verified the non-toxicity and biocompatibility of tFNA and BiRDS in vivo. All viscera samples maintained normal structures at the end of experiments.

**Method S1. Western Blotting assays**

Following treatment, the cells were harvested using whole-protein extraction kits (KeyGen Biotech Co., Ltd., Nanjing, China) to attain the total protein measurements. The target proteins were isolated using sodium dodecyl sulfate (SDS)-PAGE (10–12%). Proteins of specific molecular weights were transferred to a PVDF membrane, which was immediately blocked with bovine serum albumin (5%) for 1 h. Following incubation with primary antibodies [anti-α-SMA (1:1000), anti-E-cadherin (1:10000), anti-collagen I (1:1000), anti-fibronectin (1:1000), anti-TNF-α (1:800), anti-NLRP3 (1:1000), anti-cleaved caspase-1 (1:1000), anti-GSDMD (1:1000), anti-IL-18 (1:1000), and anti-IL-1β (1:1000)] overnight at 4 °C, PVDF membranes were incubated with the appropriate secondary antibodies (1:3000) for 1 h. The exposure signals were acquired from the gel and the blot imaging system for a subsequent semi-quantitative analysis performed in ImageJ (V1.8.0.112).

**Method S2. Statistical analysis**

All data are expressed as mean ± standard deviation. Statistical analysis was performed using Student's t-test and one-way analysis of variance. Results were considered statistically significant if the p-value was <0.05 (single-tailed: <0.05, double-tailed: <0.01, and triple-tailed: <0.001). GraphPad Prism 8 software (GraphPad Inc., La Jolla, CA, USA) was used for graphical analysis and illustrations.
